# Laparoscopic treatment of paraprostatic cyst in two dogs – complete resection, and partial resection with omentalization: a case report

**DOI:** 10.3389/fvets.2024.1270819

**Published:** 2024-04-08

**Authors:** Jiyoung Park, Heungseok Won, Gyeong Gook Park, Hee Jun Jeong, Changhwan Moon, Jaemin Jeong, Hae-Beom Lee, Dae-Hyun Kim, Seong Mok Jeong

**Affiliations:** ^1^Department of Veterinary Surgery, College of Veterinary Medicine, Chungnam National University, Daejeon, Republic of Korea; ^2^Armed Forces Medical Research Institute, Daejeon, Republic of Korea; ^3^Ulsan S Animal Medical Center, Ulsan, Republic of Korea

**Keywords:** abdominal mass, prostatic cyst, paraprostatic pseudocyst, computed tomography, magnetic resonance imaging, laparoscopic resection, omentalization, dog

## Abstract

Two intact male dogs were evaluated for symptoms, including hematuria, prostatomegaly, anuria, lethargy, and abdominal mass. Presurgical evaluations, including complete physical examinations, blood examinations, abdominal radiography with contrast (only in Case 2), ultrasonography, and computed tomography and magnetic resonance imaging (only in Case 1), were performed. A paraprostatic cyst was diagnosed initially, and laparoscopic exploration and surgery were performed. Complete resection was performed in case 1, whereas partial resection with omentalization was performed in case 2. Histopathological examination of the tissue samples confirmed the presence of paraprostatic pseudocysts in both cases, with no evidence of an epithelial lining. These two cases represent the first documented instances of laparoscopic treatment for extraparenchymal prostatic cysts. The laparoscopic treatment proved feasible even in the case of a giant cyst causing anuria (Case 2). Paraprostatic cysts should be considered a potential differential diagnosis for abnormal urination accompanied by an abdominal mass, and long-term postoperative follow-up is necessary.

## 1 Introduction

Prostatic diseases are common in older, intact male, large-breed dogs, especially in German shepherds and Doberman pinchers (1, 2). Post-mortem examinations have revealed that 75.6% of dogs dying from prostate-unrelated disorders have prostatic diseases (3). Prostatic cysts (PCs) account for 1.1–14% of all prostatic diseases (1, 3, 4), and approximately 42% of PCs become infected (1). Multiple cystic changes rather than large solitary cysts are more common (5). Intraparenchymal cysts (IPCs, intraprostatic cysts, retention cysts) originate from microcysts, resulting from the accumulation of prostatic secretions due to increased production or obstruction of ducts with prostatic hyperplasia (1). In contrast, the etiology of extraparenchymal cysts (EPCs, paraprostatic cysts; pPCs) is not fully understood, but their association with the uterus masculinus, a remnant of the Müllerian duct (paramesonephric duct), has been suggested (1, 4) in addition to the same manner as IPCs (6). pPCs vary in size, are confined within a fibrocollagenous capsule, and usually have no direct involvement of the prostatic parenchyma despite occasional connection with the prostatic capsule (1). It is craniolateral or dorsal to the prostate and could be palpable through the pelvic cavity sometimes (3). However, there is also an opinion that it does not seem plausible to distinguish these lesions based solely on their anatomical location; for all types of larger discrete cysts associated with the prostate gland, a general term of “prostatic cyst” is more appropriate (5).

Clinical signs of PCs depend on the degree of compression exerted on adjacent structures in the caudal abdomen or pelvic cavity due to lesion enlargement (1, 7, 8). It can be from asymptomatic to systemic illness in case of secondary infection; typical signs include tenesmus, dysuria, hematuria, stranguria, urinary retention/incontinence, dyschezia, ribbon-like feces, perineal hernia, inguinal hernia, abdominal enlargement, or pain (1, 3, 4, 9, 10). Ultrasonography, which shows a large anechoic structure with or without internal septa, has become the primary tool for diagnosing PCs owing to its widespread availability (1, 5, 11). Retrograde contrast radiography also allows visualization of the relative positions of the bladder, prostatic urethra, and prostate (5).

As non-invasive techniques such as aspiration or sclerotherapy with alcohol do not provide permanent resolution, they may be recommended as a temporary measure to relieve urinary retention while waiting for surgical intervention or the resolution of any accompanying azotemia (5). Surgical intervention is the definitive treatment option for large cysts or in patients with recurrence (1, 5). Complete resection is the most favorable option; however, partial resection combined with omentalization can also be performed (1). For small cysts, castration alone can cause lesions to subside, with the prostate shrinking after 3–6 weeks due to the termination of testosterone production (6).

This report describes the diagnostic procedures, laparoscopic surgical findings, and histopathological diagnosis for two types of PCs.

## 2 Case description

### 2.1 Case 1

An 8-year-old, castrated male German shepherd weighing 28.5 kg presented with an intra-abdominal mass. He had undergone castration 3 days earlier for prostatomegaly and hematuria. At the time of presentation, the patient was bright and alert, and the gross hematuria had resolved. Physical and laboratory examinations (complete blood count, chemistry, electrolytes, C-reactive protein [CRP], and urinalysis) showed no remarkable findings except high CRP (5.2 mg/dL, reference range; RR <2 mg/dL) and micro-hematuria (2+).

**Figure 1:**
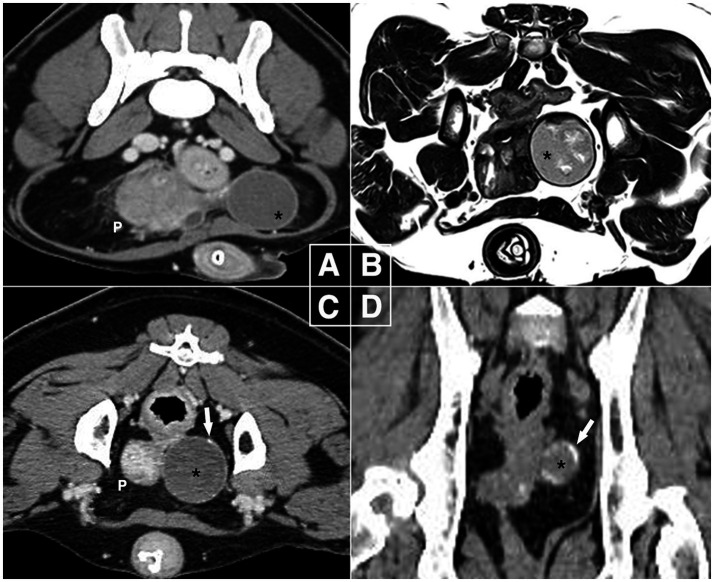
Diagnostic images in canine paraprostatic cyst (Case 1). **(A)** Computed tomography at first presentation (delayed phase, soft tissue window, transverse plane), **(B)** magnetic resonance imaging after 4 weeks (T2-weighted transverse plane), **(C,D)** one-year follow-up computed tomography (delayed phase, soft tissue window, transverse & dorsal plane). The intrapelvic mass-like lesion remained 3.5 cm in size over one-year period but developed calcifications on the capsular region by the end. The right side of the images corresponds to the left side of the patient. P, prostate; black asterisk, mass-like lesion of paraprostatic cyst; white arrow, calcified part of the lesion.

Plain radiography revealed a round soft tissue opaque mass on the left ventral side of the bladder neck, which was a cystic mass (3.5 cm) with irregular hyperechogenicity on ultrasonography. The lesion was located by the enlarged prostate; computed tomography (CT) with contrast study (Figure 1A) revealed a spherical structure with a capsular connection by a stalk but no parenchymal communication with the enlarged prostate (58 × 50 × 36 mm). No distant organs were involved. When ultrasound-guided fine needle aspirations were performed, cytological analysis for the cystic lesion revealed the presence of red blood cells, hemosiderin, and hemosiderin-laden macrophages; however, no diagnostic cells suggestive of malignancy were detected. For the prostate, on the other hand, prostatic epithelial cells lost their typical sheet-like structures with low cellularity. They exhibited mild anisocytosis, round to oval pleomorphic nuclei, and coarse chromatin within the basophilic cytoplasm, indicating neoplastic changes.

Four weeks later, magnetic resonance imaging (MRI) showed the prostate size had decreased (31 × 24 × 37 mm) with a hyperintense cystic structure measuring 10 × 3 mm, while the intrapelvic paraprostatic lesion remained unchanged (Figure 1B); it exhibited irregular, heterogeneous, and hyperintense parenchyma surrounded by a thin hypointense capsule, which still had no connection to other organs. Based on these findings, benign prostatic hyperplasia and pPC were diagnosed, and the patient was monitored by the referring veterinarian.

At a 1-year follow-up, CT showed pPC without a significant change in size or parenchymal appearance. However, there were several areas of calcification, mainly in the capsular region (Figures 1C,D). For histopathological diagnosis, elective laparoscopic surgery was performed under general anesthesia with isoflurane inhalation. Midazolam (0.2 mg/kg, IV), butorphanol (0.2 mg/kg, IV), and propofol (4 mg/kg slow IV) were administered as premedication and for induction. Cefazolin (20 mg/kg, IV) and meloxicam (0.2 mg/kg, IV) were administered for antimicrobial prophylaxis and analgesia, respectively. The dog was placed in the right lateral oblique recumbency, and the surgical table was tilted as needed in the Trendelenburg position. The video tower was placed at the caudal end of the table. The primary surgeon was on the right side of the dog, and the assistant who handled the camera stood on the left. Three 5-mm ports were placed using the Hasson technique: a primary port (cranial to umbilicus) for the telescope (5 mm, 0°, 1488 HD; Stryker, Michigan, USA), and second and third instrumental ports (bilateral paramedian cranial to prepuce). Intra-abdominal pressure was maintained at approximately 10 mmHg throughout the procedure, and the ports were used interchangeably.

**Figure 2:**
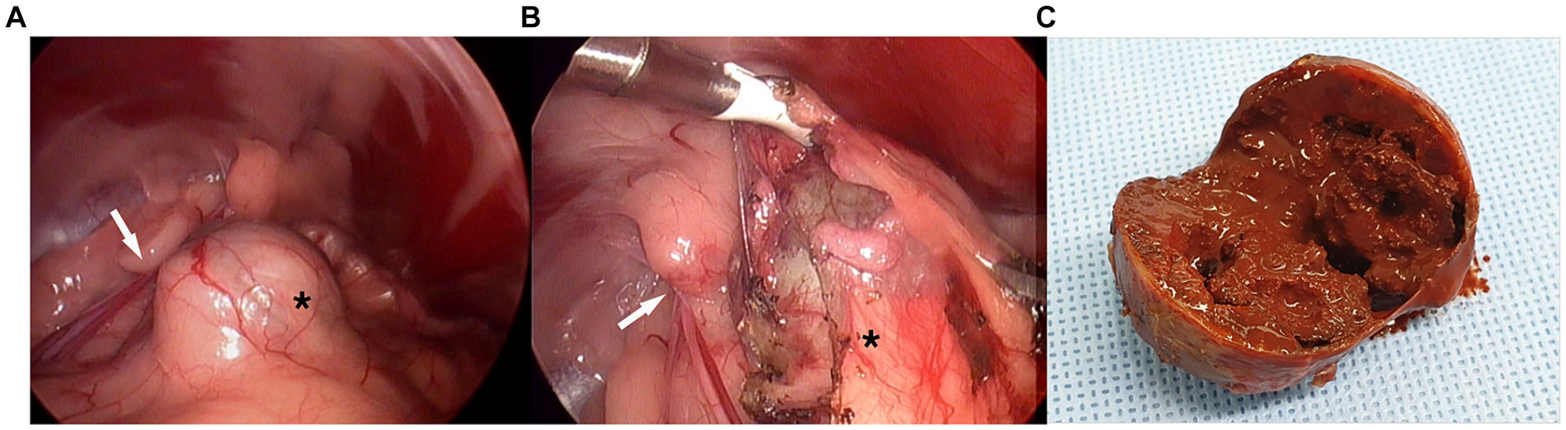
Laparoscopic images in complete excision of canine paraprostatic cyst (Case 1). **(A)** Spheric mass cranio-medial to the left internal inguinal ring, **(B)** the lesion being dissected from surrounding fat, **(C)** gross appearance of the cut surface black asterisk, mass-like lesion of paraprostatic cyst surrounded by perivesical fat; white arrow, left internal inguinal ring.

Exploration of the caudal abdomen revealed a spherical mass just cranial to the left internal inguinal ring (Figure 2A). It was surrounded by perivesical/periprostatic fat and was mobile within the fat when pushed away. The peritoneum was opened using dissecting forceps at the location of the least fat. The adipose tissue was peeled off with Maryland forceps or dissected from the mass using a vessel-sealing device (LigaSure; Medtronic, Minneapolis, MN, USA) (Figure 2B). The surface of the capsule was mildly vascularized, bluish-gray, or creamy yellow. The lesion was not specifically fixed and exhibited a rotating or rolling motion during dissection. Bleeding was minimal and well controlled by compression. The excised mass was placed in a specimen retrieval bag and retrieved through the enlarged portal site. No additional dissection was performed for comprehensive inspection or biopsy of the prostate. Portal sites were routinely closed after bupivacaine infiltration. The mass was 3.5 cm in diameter, with a thick, tight, homogeneous capsule, and had a brick-red, mushy sludge-like appearance with a necrotized area on the cut surface (Figure 2C).

**Figure 3:**
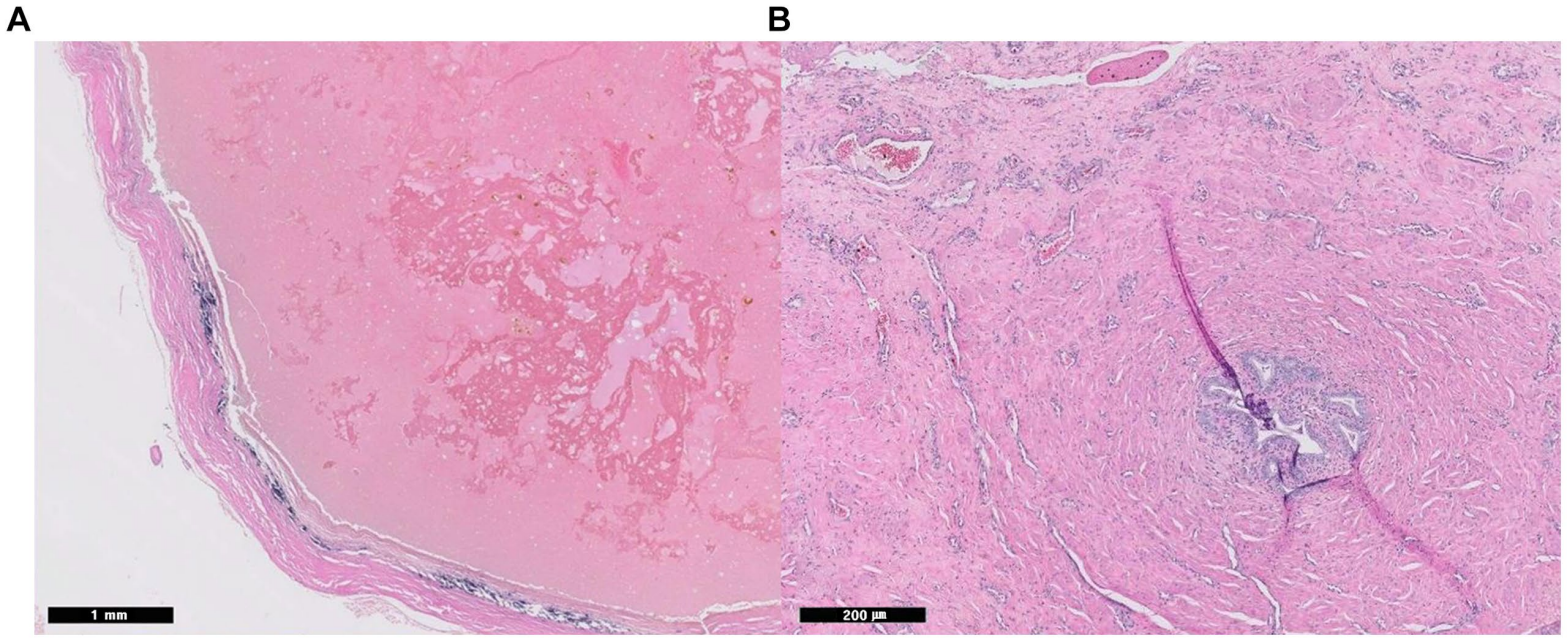
Histopathological images of paraprostatic pseudocyst in 2 dogs. Two intrapelvic cystic lesions were confirmed as pseudocysts histopathologically with an absence of epithelial lining (Hematoxylin and eosin staining, **A**; case 1, x 20, **B**: case 2, x 100). In case 2, small foci of glandular ductules represent prostatic glandular tissue **(B)**.

The patient recovered uneventfully and was discharged on postoperative day (POD) 1 with a good appetite and normal activity. Histopathological examination confirmed the lesion was a paraprostatic pseudocyst (Figure 3A). The capsule had a thick band of fibrous connective tissue containing fibroblasts and multiple areas of mineralization but no epithelial lining. No neoplastic cells, inflammation, or infectious agents were observed. The patient had been doing well without any issues regarding urination or recurrence of clinical signs. Eight months postoperatively, however, he died of an unrelated cause, systemic lupus erythematosus.

### 2.2 Case 2

**Figure 4:**
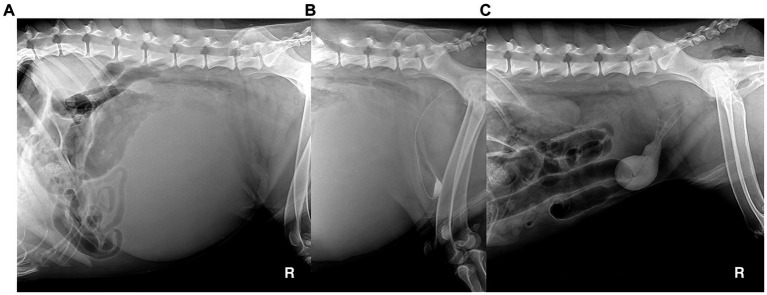
Radiographic images of canine paraprostatic cyst (Case 2) before (**A**: plain radiograph, **B**: retrograde contrast cystogram) and after percutaneous aspiration of the cyst (**C**: retrograde contrast cystogram).

A 5-year-old, intact male dog weighing 40 kg presented with lethargy, severe depression, and had astasia, abdominal enlargement, and anuria lasting >24 h. Plain radiography revealed a giant round soft tissue opaque mass that deviated intestines cranially (Figure 4A) and measured 32 cm in diameter. On ultrasonography, the lesion was a cyst, not a parenchymal mass. A retrograde positive contrast cystogram showed a compressed urinary bladder being flexed to the caudoventral direction in isolation from the mass (Figure 4B). Ultrasound-guided percutaneous cyst aspiration was performed, and > 4 L of turbid brownish fluid was removed. The lesion was suspected to be a pPC with a thickened wall (>1 cm). There were no specific findings in the urinary system except for prostatomegaly (7 cm) and a dilated urethra up to the mid-part of the prostatic urethra. No direct communication between the urethra and cyst was identified on cystourethrography (Figure 4C). Blood levels of urea nitrogen, creatinine, phosphorous, and electrolytes were 30 mg/dL (RR: 7–27 mg/dL), 1.3 mg/dL (RR: 0.5–1.8 mg/dL), 7 mg/dL (RR: 2.5–6.8 mg/dL), 3.6 mmol/L (potassium, RR: 3.9–5.1 mmol/L), respectively. The aspirate contained only neutrophils with no other remarkable finding; the creatinine level was 0.9 mg/dL. The owners did not provide consent for CT or an exploratory open celiotomy, agonizing over euthanasia. Voluntary urination was identified after 14 h with dribbling and hematuria, and the cyst re-filled quickly and enlarged to more than 10 cm in diameter the next day.

Three days later, laparoscopic exploration and partial resection of the pPC with omentalization were performed under general anesthesia, as described in case 1. The patient was in left lateral recumbency with the right hind limb abducted and the stifle flexed. The primary surgeon was positioned on the ventral side of the patient, and the assistant stood on the right side of the primary surgeon. Three 5-mm ports were placed using the Hasson technique: a primary port (cranial to umbilicus) for the telescope (5 mm, 0°, 1488 HD; Stryker, Michigan, USA) and second and third instrumental ports (right paramedian caudal to primary port, caudal to umbilicus) for the instruments. The surgical table was adjusted as needed in the Trendelenburg position, and the intra-abdominal pressure was maintained at approximately 10 mmHg throughout the procedure.

**Figure 5:**
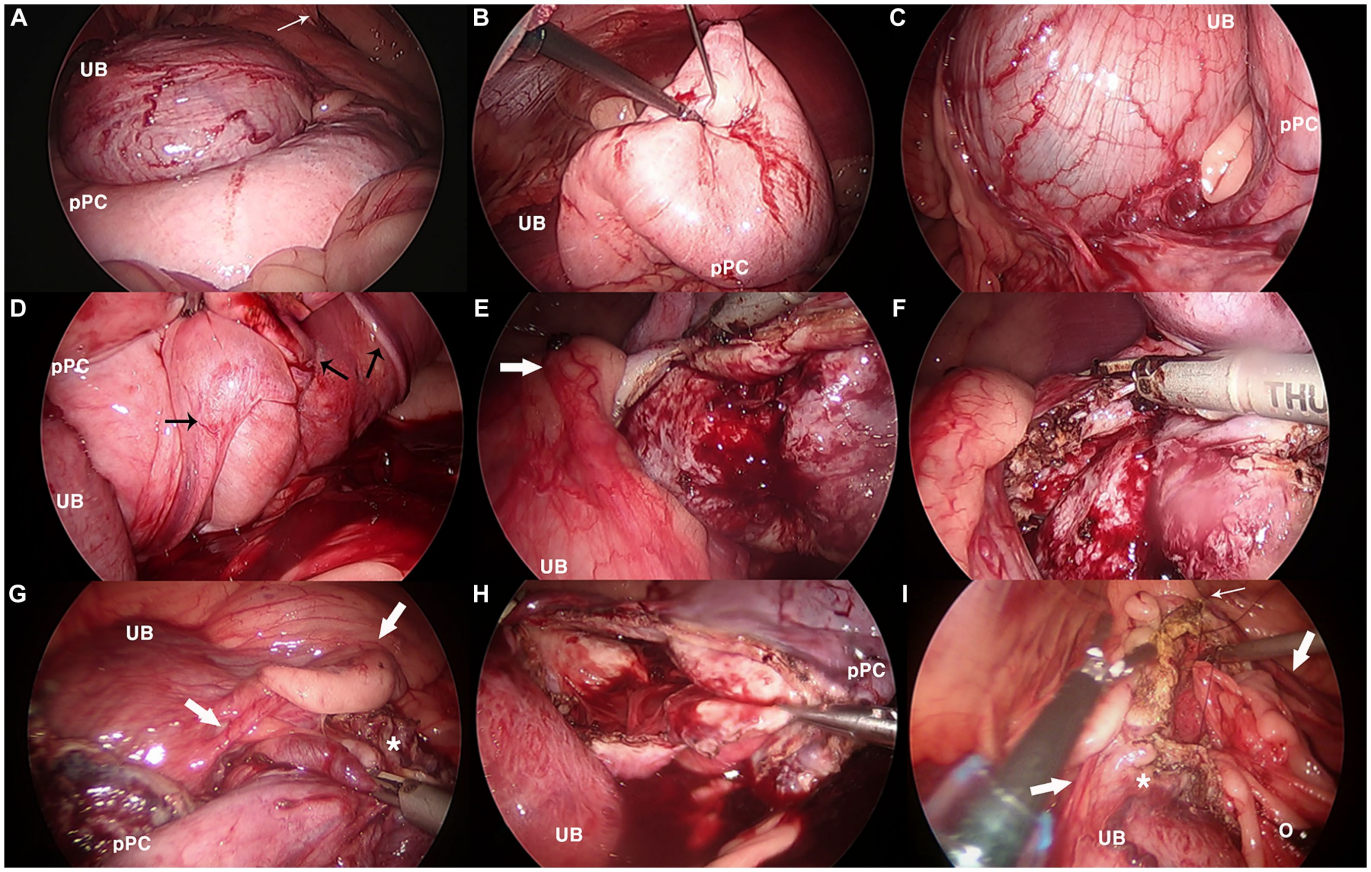
Laparoscopic images in partial resection and omentalization of canine paraprostatic cyst (Case 2). **(A)** Urinary bladder (UB) and dorsally located paraprostatic cyst (pPC) under initial exploration, **(B)** cystic lesion being aspirated with first percutaneous anchoring suture, **(C)** neurovascular pedicle between the urinary bladder and the cyst, **(D)** vas deferens (black arrows) running dorsal surface of the cyst, **(E–G)** thick, easily bleeding cystic wall being resected with vessel sealing device, notice the right ureter (thick white arrow), **(H)** paraprostatic cyst after resection completed, **(I)** intracorporeal suturing between omentum (o) and remaining cuff of the cystic stump (*). Thin white arrow, right internal inguinal ring.

When the caudal abdomen was explored, a pale-pink balloon-like lesion located dorsal to the bladder exhibited a smooth surface (Figure 5). The cyst was heavy, with a thick and tough wall, making manipulation demanding. It was bordered by the right vas deferens and ureter at the cystic root level. The right vas deferens was identified on the medial side of the lesion, which ran along the surface of the pPC root from the medial to the dorsal and lateral sides, escaping through the right inguinal ring. The ureter was located ventral and lateral to the lesion. The cystic wall bled to instrumental grasping moderately. The cyst was anchored to the abdominal wall, and percutaneous aspiration was performed using a Veress needle. When opened with a vessel-sealing device (Thunderbeat; Olympus, Tokyo, Japan), blood-tinged fluid leaked into the abdominal cavity. An additional anchoring suture was placed, and the wall was excised from the dorsal wall. Vascularity was prominent inside the wall, and bleeding occurred frequently on the cut surface. The excision was continued on the ventral side after the anchoring sutures were released. Once the resection was completed with a cuff of less than approximately 3 cm, the omentum was packed into the inner space of the stump and fixed to the cuff with 3–0 polydioxanone using intracorporeal sutures. No active bleeding was observed when the surgical site was re-examined after lavage. The resected pPC was retrieved through one of the midline ports enlarged by an additional incision, and the portal sites were closed routinely after bupivacaine infiltration. Castration was performed, and the patient recovered without any anesthetic complications. A bacterial culture of the aspirate revealed no bacterial growth.

On POD 6, the patient’s urination returned to normal, and he was discharged. Histopathological examination revealed a paraprostatic pseudocyst without an epithelial lining. However, there were small foci of glandular ductules bordered by smooth muscle, which may represent prostatic glandular tissue (Figure 3B). When the patient returned on POD 18, he was bright, alert, and had a good appetite and normal activity. There were no issues with urination, defecation, or dribbling. Ultrasonography revealed no remarkable findings in the urinary system but prostatomegaly. The omentum maintained their intended positions without ascites. Since then, the patient has escaped follow-ups.

## 3 Discussion

pPCs (EPCs) are uncommon, with large and solitary cases being even rarer (2, 5, 12, 13). Nevertheless, a recent canine study involving 44 sterile PCs—comprising 29 EPCs (65.9%), 11 IPCs (25.0%), and 4 concurrent cysts (9.1%)—revealed that ultrasonographically large cysts measuring over 20 cm accounted for 52.0% of the 17 EPCs and 16.7% of the 6 IPCs, respectively (1). Although cystic lesions in the present cases were consistently referred to as pPCs, Case 2 highlights that conventional classification of PCs based on their location may not always be significant, as previously mentioned. Initially categorized as a pPC (EPC), Case 2 was later found, upon histopathological examination, to have glandular ductules in the resected cystic wall, raising suspicion that it might be an IPC, potentially indicating that the lesion is a part of the prostate gland itself. This also demonstrates the possibility that prolonged massive fluid accumulation could cause an IPC to stretch, protrude, and deviate, resembling an EPC. Accordingly, the term of PC was also used interchangeably in the following discussion as a comprehensive meaning. Meanwhile, pseudocysts are distinguished from true cysts, which possess an epithelial-lined capsule, through histopathological examination, where the absence of such lining is observed. Clinically, these lesions may be initially regarded as cysts, but upon histopathological examination, they are confirmed as pseudocysts due to the absence of an epithelial lining.

Boland et al. reported that ultrasound-guided percutaneous drainage was a valuable alternative to surgery for prostatic abscesses and PCs in 13 dogs (8). However, that study repeated drainage up to four times under anesthesia or sedation. Moreover, the lesions were relatively small, measuring a maximum of 6.5 cm in size, and castration was performed on intact males (*n* = 10) as part of the treatment approach. In Case 1, the pPC was small but led to surgical resection because castration only resulted in prostatic shrinkage, not cystic resolution. Progressive changes in capsular mineralization needed to be examined histopathologically.

Although mineralization is mentioned to be uncommon (3, 10), this finding would be more common than indicated in the literature. It is thought to be characteristic appearance of the lesion, and neoplasia is less likely, in contrast to intraparenchymal prostatic mineralization in castrated dogs (5). The capsule undergoes ossification and mineralization even though it is not a neoplastic change (3, 10, 14); it would be a process of chronicity, as seen in Case 1. Moreover, giant pPC containing cauliflower-like metaplastic ossification has also been reported (15).

For PCs, the surgical approach, procedure choice, and resection extent depend on the lesion’s location, size, and degree of adhesion to the surrounding structures (1, 2, 16). Previous techniques of passive drain application, marsupialization, and partial prostatectomy are no longer recommended (1). Complete or partial resection and omentalization with open celiotomy provided postoperative remission of clinical signs in 88.6% of 44 patients, with a surgical success rate of 80% for EPCs and 100% for IPCs (1). Postoperative complications included urinary incontinence (15.9%; transient, 11.3%; and permanent, 4.5%), transient urinary tract obstruction (6.8%), urinary retention (4.5%), dysuria (2.3%), EPC recurrence (4.5%), and death (6.8%). Excessive dissection along the dorsal and lateral sides of the bladder, prostate, and urethra may lead to detrusor atony, incontinence, or bladder ischemia (13).

Regarding laparoscopic procedures for canine PCs, as of our latest knowledge, only two reports have been published to date (9, 17), one of which was published in the English literature; partial resection and omentalization for 3 cm PC in an intact male French bulldog (17). A previous study on laparoscopy-guided prostate biopsy emphasized the procedure’s safety, as laparoscopy allows complete visualization and the ability to convert to conventional open surgery if necessary (18). The two PCs in this report were intra-abdominal, and there were no space issues during the laparoscopic procedure. Both patients were large breed dogs, and instrumental and gravitational retraction, in addition to pneumoperitoneum, created a sufficient working space. Moreover, the magnification and light of laparoscopy allow for good visualization.

The procedure for complete resection in Case 1 was relatively straightforward due to the small, laterally located lesion. In contrast, in Case 2, exploration of the intrapelvic area and handling of the cyst were challenging due to its size, weight, location dorsal to the bladder, and thick cystic wall that bled easily. However, marionette or anchoring sutures offset the weight of the cyst as well as requirement of additional portal placement. Nonetheless, using larger, robust instruments, such as 10 mm forceps with toothed jaws, could have been beneficial. An open celiotomy might have been an appropriate approach initially; however, the laparoscopic procedure was completed successfully without conversion.

While the laparoscopic procedure for PC itself may be simple, the most crucial aspect to consider is its relationship with the surrounding structures, primarily due to the lack of tactile feedback. Preoperative preparation, including voiding the urinary bladder and aspirating the cyst as much as possible in case of partial resection, helps minimize contamination of the surgical field. It also ensures a spacious and clear working space with improved visualization, ultimately leading to a reduction in surgical time. In complicated lesions such as Case 2, a preoperative CT/MRI examination could be beneficial for surgeons to better understand the configuration of the lesion and its relation to the surrounding structures.

In humans, cases of prostatic utricle cysts (congenital anomalies derived from the Müllerian duct remnant) treated with laparoscopic excision without omentalization have been reported (19–23). Sixteen patients showed no complications or recurrence, but there was a disastrous case of accidental cystectomy with bilateral ureteral injury (24). Given the routine use of vessel-sealing devices in laparoscopic surgery, it is critical to make great efforts to avoid tissue trauma. LigaSure can not only cause direct damage but also generates thermal energy during dissection close to the ureter, inflicting thermal injury. This can result in later necrosis, consequent perforation, with urinary ascites identified as an acute finding (25). Stricture formation, hydronephrosis, and renal loss are the most significant long-term complications of ureteral injury (26). To prevent iatrogenic damage, a cystourethroscopic guiding light, cannulation of ureters with ureteral catheters, or real-time visualization of ureter using fluorescent ureterography with indocyanine green helps differentiate the ureters intraoperatively (22, 23, 27). Moreover, in partial resection and omentalization, as seen in Case 2, leaving the cuff of the stump sufficiently would enable surgeons to resect the lesion with greater confidence, which allows for sparing the nearby ureter and rectum, as well as the neurovascular pedicles.

In the present cases, cytological or histopathological examination of the prostate was not performed. However, needle aspiration or incisional biopsy is always recommended, even in the absence of gross neoplastic infiltration (5). Furthermore, regular long-term monitoring should be maintained, as the later development of prostatic abscess or cystadenocarcinoma at the original surgical site (12) or abdominal metastasis (carcinomatosis) of prostatic carcinoma along the omental graft stalk following previous omentalization has been reported (28), in addition to cystic recurrence.

In conclusion, laparoscopic treatment, whether through complete or partial resection with omentalization, is feasible for canine pPCs, even in large symptomatic cases. Early surgical intervention at the stage of a small, simple cyst rather than a complicated large cyst simplifies the surgery in terms of both anesthetic time and damage to surrounding vital tissue, which should decrease the chance of postoperative complications, eventually.

## Data availability statement

The original contributions presented in the study are included in the article/supplementary material, further inquiries can be directed to the corresponding authors.

## Ethics statement

Ethical approval was not required for the study involving humans in accordance with the local legislation and institutional requirements. Written informed consent to participate in this study was not required from the participants or the participants’ legal guardians/next of kin in accordance with the national legislation and the institutional requirements. Ethical approval was not required for the studies involving animals in accordance with the local legislation and institutional requirements because this is not a research paper but a case report. Written informed consent was obtained from the owners for the participation of their animals in this study.

## Author contributions

JP: Conceptualization, Data curation, Investigation, Writing – original draft. HW: Conceptualization, Data curation, Writing – original draft, Writing – review & editing. GP: Formal analysis, Writing – original draft. HJ: Writing – review & editing. CM: Data curation, Formal analysis, Writing – review & editing. JJ: Writing – review & editing, Formal analysis. H-BL: Writing – review & editing. D-HK: Funding acquisition, Supervision, Writing – review & editing. SJ: Funding acquisition, Writing – review & editing, Supervision.
